# Agriophyllum Oligosaccharides Ameliorate Diabetic Insulin Resistance Through INS-R/IRS/Glut4-Mediated Insulin Pathway in db/db Mice and MIN6 Cells

**DOI:** 10.3389/fphar.2021.656220

**Published:** 2021-08-23

**Authors:** Shuyin Bao, Xiuzhi Wang, Sung Bo Cho, Yan-Ling Wu, Chengxi Wei, Shuying Han, Liming Bao, Qiong Wu, Wuliji Ao, Ji-Xing Nan

**Affiliations:** ^1^ Key Laboratory for Traditional Chinese Korean Medicine of Jilin Province, College of Pharmacy, Yanbian University, Yanji, China; ^2^ Medical College, Inner Mongolia University for Nationalities, Tongliao, China; ^3^ Department of Medicines and Foods, Tongliao Vocational College, Tongliao, China; ^4^ The Research Institute of Traditional Mongolian Medicine Engineering Technology, Tongliao, China; ^5^ College of Traditional Mongolian Medicine, Inner Mongolia University for Nationalities, Tongliao, China; ^6^ Basic Medical College, North China University of Science and Technology, Tangshan, China; ^7^ Department of Cardiology, Tongliao Second People’s Hospital, Tongliao, China; ^8^ Clinical Research Center, Yanbian University Hospital, Yanji, China

**Keywords:** Agriophyllum oligosaccharides (AOS), T2DM, insulin resistance, INS-R/IRS/Glut4 insulin pathway, db/db mice

## Abstract

We have previously reported that Agriophyllum oligosaccharides (AOS) significantly enhance glycemic control by increasing the activation of insulin receptor (INS-R), insulin receptor substrate-2 (IRS-2), phosphatidylinositol 3 kinase (PI3K), protein kinase B (AKT), peroxisome proliferator-activated receptor (PPAR)-γ, and glucose transporter 4 (Glut4) proteins in hepatic tissues. However, the effect of glucose control by AOS on the regulation of pancreatic tissues in db/db mice and MIN6 cells remains to be determined. An oral dose of AOS (380 or 750 mg/kg) was administered to type-2 diabetic db/db mice for 8 weeks to determine whether AOS regulates glucose by the INS-R/IRS/Glut4-mediated insulin pathway. Meanwhile, the effects of AOS on glucose uptake and its related signaling pathway in MIN6 cells were also investigated. The results showed that the random blood glucose (RBG) level in the AOS-treated group was lower than that in the control group. AOS reduced the levels of glycated hemoglobin (HbA1c) and free fatty acid (FFA) and significantly improved the pathological changes in the pancreatic tissues in db/db mice. Moreover, immunohistochemical analysis revealed that the expression of INS-R, IRS-1, IRS-2, and Glut4 was increased in the AOS-treated group than in the model group. Further, *in vitro* experiments using MIN6 cells showed that AOS regulated INS-R, IRS-1, IRS-2, and Glut4 protein and mRNA levels and attenuated insulin resistance and cell apoptosis. The results of both *in vitro* and *in vivo* experiments were comparable. Ultra-performance liquid chromatography coupled with time-of-flight mass spectrometric analysis of AOS with precolumn derivatization with 3-amino-9-ethylcarbazole (AEC) tentatively identified five types of sugars: glucose, lactose, rutinose, glucuronic acid, and maltotriose. Our present study clearly showed that AOS is efficacious in preventing hyperglycemia, possibly by increasing insulin sensitivity and improving IR by regulating the INS-R/IRS/Glut4 insulin signal pathway. Therefore, AOS may be considered as a potential drug for diabetes treatment.

## Introduction

Diabetes mellitus (DM), characterized by hyperglycemia, is the most common chronic metabolic disorder globally (Kim et al., 2020). Abnormal glucose levels in DM usually result from insulin resistance (IR) (Kumar et al., 2019). Currently, the number of people with DM and its complications is increasing globally (Vieira et al., 2019). The number of adult diabetic patients could increase from 425 million aged 20–79 years in 2017 to 693 million by 2045 (Cho et al., 2018; Abdel-Moneim et al., 2020). There are two types of DM: type 1 DM (T1DM) and T2DM, and the latter accounts for more than 90% of all DM cases (Nederstigt et al., 2016). The pathogenesis of T2DM is still not clear, but it is widely recognized to be related to genetic mutations, islet oocyte loss, gut microbiota changes, and IR. IR is particularly the main driver of T2DM and causes DM-related complications (Wang et al., 2019a; Gao et al., 2019). To control T2DM, developing safe, effective, and low-toxicity drugs that ameliorate IR is important. Currently, biguanides (Han et al., 2018), alpha-glucosidase inhibitors (Moelands et al., 2018), thiazolidinediones (Segawa et al., 2018), and sulfonylureas (Chen L. et al., 2017) are used to treat T2DM. However, a previous report showed that synthetic drugs are associated with side effects (Wang et al., 2019b). Metformin is generally used as an oral antidiabetic agent. Around 20–30% of patients receiving metformin tend to develop loss of appetite, nausea, vomiting, abdominal pain, acid reflux, and other gastrointestinal side effects, whereas some others develop lactic acidosis (Dujic et al., 2016). However, a previous clinical study has reported that the incidence of lactic acidosis is very low in patients with T2DM receiving metformin (DeFronzo et al., 2016). Sulfonylureas cause weight gain and patients can be at a high risk of developing serious hypoglycemia due to hyperinsulinemia (Apovian et al., 2019). Therefore, identifying novel herbal agents with fewer side effects for treating T2DM is imperative.

In China, a large number of herbs or their extracts are used to treat diseases. The Mongolian medicinal plant *Agriophyllum squarrosum*, whose medicinal properties were first recorded in Ren Yao Bai Jing Jian, is the primary ingredient of the dried above-ground biomass of *A. squarrosum* (L.) Moq. It mainly exists in China and is primarily distributed in the Northeast, North, Northwest, Henan, and Xizang regions, as well as in Mongolia and Russia among other countries. *A. squarrosum* contains a variety of biologically active constituents such as flavonoids, coumarin, oleanopane-type triterpenoid saponins, sterols, alkaloids, fatty acids, and polysaccharides (Gong et al., 2012; Li et al., 2012; Zhou et al., 2012; Ling et al., 2018). Ancient Chinese books have recorded that *A. squarrosum* can reduce the body heat, has detoxifying and diuretic properties, and is used in Chinese Mongolian medicine to treat oral ulcers, jaundice, and diabetes (Liu et al., 2013; Bao et al., 2018; Saqier et al., 2019). Moreover, modern pharmacological studies have shown that Agriophyllum oligosaccharides (AOS) play a major role in regulating blood lipid levels, decreasing blood glucose levels, and ameliorate hepatic injury in T2DM db/db mice (Bao et al., 2016a; Bao et al., 2018; Saqier et al., 2019). Nevertheless, the underlying antidiabetic mechanisms of AOS are not clear. Bao et al. (Bao et al., 2020) reported that AOS increases glucose uptake by upregulating the expression of membrane glucose transporter type 4 (Glut4), INS-R, and IRS-2 in the liver.

Insulin receptor substrate (IRS) proteins, which play a significant role in insulin signaling pathways, are cytoplasmic receptors (Ijaz et al., 2019). When insulin binds to its receptor on the cell surface, docking proteins such as IRS-1 are phosphorylated and phosphatidylinositol 3-kinase (PI3K) is activated (Deng et al., 2020). However, higher concentrations of insulin lead to the activation of IRS-2 (Ijaz et al., 2019). One of these responses in hyperglycemia leads to the activation of the Glut4 receptor, resulting in an increased uptake of glucose by cells (Hou et al., 2019; Li et al., 2021). However, whether AOS alleviates glucose uptake via these signaling pathways remains clear. Therefore, the molecular mechanisms of AOS action against diabetes still need to be determined.

Therefore, in this study, we evaluated the effects of AOS on body mass, random blood glucose (RBG), IR, and pathological changes in the pancreatic tissues of T2DM db/db mice. Moreover, MIN6 islet beta cells were used to determine the effects of AOS on cell proliferation, apoptosis, and insulin secretion, to elucidate the possible role of AOS in hypoglycemia.

## Materials and Methods

### Reagents

All chemicals and solvents in the experiments were of analytical grade. AOS, which were extracted from the *A. squarrosum* (L.) Moq. (the number of Virtual Herbarium in Chinese is HIMC0039313) and Metformin tablets (National Drug Approval no. H12020797), were supplied by the Research Institute of Traditional Mongolian Medicine Engineering Technology (Inner Mongolia, China) and Tianjin Pacific Chemical and Pharmaceutical Co., Ltd. (Tianjin, China), respectively. A free fatty acid (FFA) determination kit (Lot no. 20151201A), an insulin determination kit (Lot no. 20151101A), and glycosylated hemoglobin determination kit (hemoglobin A1c [HBA1c]; Lot no. 20151129A) were provided by the Beijing Kainuo Spring Biotechnology Co., Ltd. (Beijing, China). Rabbit monoclonal antibodies for IRS-1 (Cat no. ab52167), IRS-2 (Cat No. ab3690), insulin receptor (INS-R) (Cat no. ab60946), and Glut4 (Cat no. ab654) were provided by Abcam Inc. (Burlingame, CA, United States). Rabbit monoclonal antibodies for GAPDH antibody (Cat no. GTX627408) were provided by GeneTex (Irvine, CA, United States). Goat anti-rabbit IgG (Cat no. 100995) was provided by Sunrise Services Inc. (Gaithersburg, MD, United States). The Kits of Revert Aid First Strand cDNA Synthesis Kit (Batch no. 00285300) and Platinum SYBR Green qPCR Super Mix-UDG with ROX (Batch no. C11744-100) were provided by the Thermo Fisher Scientific (Waltham, MA, United States). RPMI-1640 cell culture medium (Lot no. 8116118) and fetal bovine serum (FBS) (Lot no. A79E00G) were supplied by the Gibco Company (Grand Island, NY, United States). Cell Counting Kit-8 (CCK-8) was supplied by the Dojindo Laboratories (Kyushu Island, Japan). Reference standards (purity >98%) of glucose and 3-amino-9-ethylcarbazole (AEC) were provided by the Shanghai Tauto Bio-Technology Co., Ltd. (Shanghai, China). High-performance liquid chromatography-grade acetonitrile was purchased from Fisher Scientific (Fair Lawn, NJ, United States). The experiments used purified water produced from the Milli-Q Water Purification System (Millipore, Milford, MA, United States).

### Extraction of AOS

The extraction and purification of the AOS were performed as described in our previous research (Bao et al., 2019; Bao et al., 2020).

### *In Vivo* Studies

#### Experimental Design and Animal Treatment

Eight-week-old db/db male mice and nondiabetic control db/m male mice were provided by Cavens Lab Animal Co., Ltd. (Changzhou, China, approval no. SCXK (Su) 2001-0003) and caged in an automatically controlled animal facility in a 12-h light/dark cycle and at 22°C ± 2°C and 40–70% relative humidity. All the animals were freely allowed access to food and water. After a week of adaptive feeding, the diabetic model was established with an RBG level of more than 16.7 mmol/L. Then, the db/db mice were randomly allocated to four groups (n = 10 per group): 1) model group (Model), in which the mice were intragastrically administered with distilled water; 2) low-dose AOS group (LAOS), in which the mice were intragastrically administered with AOS at a dose of 380 mg/kg/day for 8 weeks; 3) high-dose AOS group (HAOS), in which the mice were intragastrically administered with AOS at a dose of 750 mg/kg/day for 8 weeks; and 4) metformin group (MET), in which the mice were intragastrically administered with metformin at a dose of 160 mg/kg/day for 8 weeks. The dose of AOS was determined in our previous study (Bao et al., 2016a). Ten db/m mice were treated with a vehicle in the control group (Control), and all the groups were treated at 9 a.m. daily. At the end of the experiments, all the mice were sacrificed under anesthesia after the blood and pancreas were sampled.

**FIGURE 1:**
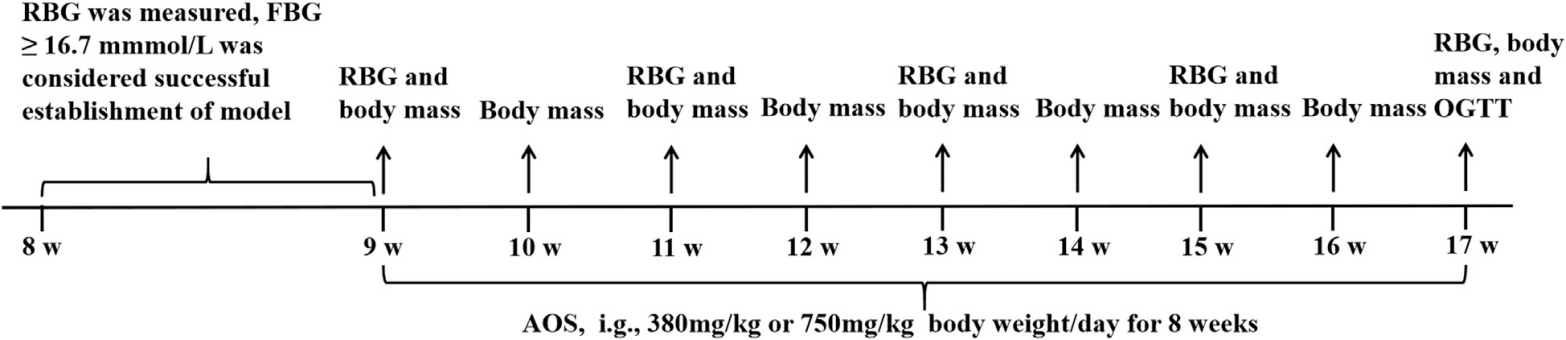
Experimental design of the *in vivo* study. The db/m and db/db mice were housed at 22 ± 2°C, 40–70% relative humidity and 12-h light/dark cycle. After 7 days, RBG was measured by snipping the tails. The diabetic mice were randomly divided into Model, AOS (380 or 750 mg/kg/day), and Metformin (160 mg/kg/day) groups. The drug treatment lasted 8 weeks. RBG levels were measured weekly during 8 weeks of AOS and metformin treatment. At the end of the experiments, the mouse were sacrificed for further experiments.

All the experimental procedures were performed by following the Guide for the Care and Use of Laboratory Animals approved by the Institutional Animal Care and Use Committee of Medical College, Inner Mongolia University for the Nationalities (Ethics committee no: 2017-9-10-01). Every effort was made to minimize the number of animals used and their suffering. The experimental design is described in Figure 1.

### Random Blood Glucose

RBG was estimated by the tail snipping method. The blood glucose levels were measured (30 min after dosing) using a glucose meter at 9, 11, 13, 15, and 17 weeks.

### FPG, FINS, and HOMA-IR

The fasting plasma glucose (FPG) level from 12-h fasted mice was measured from the eyeball 17 weeks after administering the injection. The fasting serum insulin levels (FINS) were quantified by using an enzyme-linked immunoassay (ELISA) kit (Keno Spring Biotechnology Co., Ltd., Beijing, China). The homeostasis model assessment-IR (HOMA-IR) index was calculated as previously described (Hsu et al., 2013) using the following equation: FPG (mmol/L) × FINS (mU/L)/22.5.

### Biochemical Determinations

The levels of advanced glycation end products (AGEs) and FFA were estimated with an ELISA kit (RENGEN Bioengineering Technology Co., Ltd., Beijing, China), and the glycated hemoglobin (HbA1c) level was detected in the blood drawn via a tail prick using HbA1c monitors (NycoCardREADER2; Axis-Shield Diagnostics Co., Ltd., Scotland, United Kingdom) at 17 weeks of injection.

### Histological Analysis of the Pancreatic Tissues

After the animals were sacrificed, their excised pancreas was first rapidly fixed in 2.5% glutaraldehyde for 4 h and then fixed in 1% osmic acid for 1 h. After dehydration of the tissues with ethanol, the tissues were covered with epoxy resin and cut into ultrathin sections, followed by staining with lead citrate. Then, transmission electron microscopy (Hitachi, Japan) was used to observe the pancreas’ ultrastructural changes. The remaining tissues were fixed in 4% paraformaldehyde, embedded in paraffin, and then cut into 4-μm-thick sections and stained with HE or Masson trichrome staining. Finally, the stained section was examined for its morphological structure by a microscope (OLYMPUS, Tokyo, Japan).

### Immunohistochemistry Analysis of INS-R, IRS-1, IRS-2, and Glut4.

The pancreatic tissues embedded in paraffin were dewaxed in xylene and rehydrated in gradient alcohol. After antigen retrieval and hydrogen peroxide (H_2_O_2_) blocking, the sections were next incubated with a primary antibody against INS-R, IRS-1, IRS-2, and Glut4. The positive expression was evaluated by using diaminobenzidine (ZSGB-BIO, Beijing, China) after incubation with the secondary antibody. Fluorescent images were observed and photographed by using the Bio-Rad Radiance 2,100 Confocal Microscope (Bio-Rad, Hercules, CA, United States).

### *In Vitro* Studies

#### Cell Proliferation Assay

MIN6 pancreatic beta cells (Cell bank of Fudan University, Shanghai, China) were cultured in RPMI-1640 medium. The cells were seeded in a 96-well plate. Each well contained 1 × 10^5^ cells and was treated with various concentrations of AOS from 0 to 4,000 μg/ml. For assays using glucose-induced cells, the cells were washed twice with phosphate-buffered saline (PBS) and incubated with normal (5.5 mM) or high (33.3 mM) glucose concentrations for 24 h. Thereafter, the cells were cultured in different concentrations of AOS (0, 16, 32, and 64 μg/ml) for 24 h. The cells treated with a normal glucose concentration (5.5 mM) and metformin (32 μg/ml) were considered negative and positive controls, respectively. Cell viability was estimated using the CCK-8 Kit in accordance with the manufacturer’s instructions.

### Insulin Secretion

MIN6 cells of 80% confluency in 48-well plates were cultured in RPMI-1640 medium for 24 h. The growth medium was removed on the day of the trial, after which a 5.5- or 33.3-mM glucose-containing medium was added to each well. After 24 h, the supernatant was collected, and the cells were stimulated with AOS (16 or 32 μg/ml) or metformin (32 μg/ml) for 60 min. The level of insulin secretion in the supernatant and cells were estimated using the Mouse Ultrasensitive Insulin ELISA Kit (Keno Spring Biotechnology Co., Ltd., Beijing, China).

### Analysis of Annexin V-FITC/Propidium Iodide Flow Cytometry

Annexin V-FITC binding and propidium iodide (PI) staining were performed using flow cytometry (Becton Dickinson) according to the manufacturer’s protocol (Becton Dickinson Franklin Lakes, NJ, United States). Briefly, MIN6 cells were seeded in six-well plates and cultured for 24 h, pretreated with or without glucose (33.3 mmol/L) for 24 h, and then exposed to AOS (16, 32, and 64 μg/ml) for 24 h. Then, the supernatant was collected, and the attached cells were trypsinized and collected. The cells were washed twice with ice-cold PBS, resuspended in binding buffer, stained with annexin V-FITC and PI for 15 min, and incubated in the dark. Finally, a flow cytometer was used to analyze the samples. The total percentage of apoptotic cells was determined as the sum of the percentages of both early and late apoptotic subpopulations.

### Quantitative Real-Time PCR

Total RNA was extracted from MIN6 cells using the TRIzol reagent (Invitrogen, Carlsbad, CA, United States) and used to synthesize the first-strand cDNA using the RevertAid First-Strand cDNA Synthesis Kit (Thermo Fisher Scientific). Quantitative real-time PCR (qRT-PCR) was performed using the Real-Time PCR Detection System (Bio-Rad). The specific primer sequences are summarized in Table 1. The results of three independent experiments performed for determining INS-R, IRS-1, IRS-2, and Glut4 mRNA levels were normalized against β-actin levels for each sample.

**TABLE 1:** Sequences of the primers used for polymerase chain reaction.

Gene	Forward	Reverse	Product size (bp)
INS-R	5′-GTG​CTG​CTC​ATG​CCC​TAA​GA-3′	5′-AAT​GGC​CTG​TGC​TCC​TCC​TG-3′	234
IRS-1	5′-GGA​AGA​GAC​TGG​CAC​TGA​GG-3′	5′-CTG​ACG​GGG​ACA​ACT​CAT​AT-3′	199
IRS-2	5′-GGC​TTC​CAG​AAT​GGT​CTC​AA-3′	5′-AAG​TCA​ATG​CTG​GCG​TAG​GT-3′	239
Glut-4	5′-TTC​CTT​CTA​TTT​GCC​GTC​CTC-3′	5′-TCT​GGC​CCT​AAG​TAT​TCA​AGT​TCT-3′	170
β-actin	5′-TCC​ATC​ATG​AAG​TGT​GAC​G-3′	5′-GTA​CTT​GCG​CTC​AGG​AGG​A-3′	171

### Western Blot Analysis

Total protein was extracted from MIN6 cells with cold RIPA lysis buffer containing proteases and separated by sodium dodecyl sulfate-polyacrylamide gel electrophoresis, followed by electrotransfer onto a PVDF membrane (Millipore, Billerica, MA, United States) and immunoblotting. Then, the membrane was blocked in 5% skim milk in Tris-buffered saline containing 0.1% Tween-20 for 1 h and incubated overnight at 4°C with anti-Glut4, anti-INS-R, anti-IRS-1, anti-IRS-2, and anti-GAPDH primary antibodies (1:1,000; Abcam, Cambridge, MA, United States). The membranes were washed three times in Tris-buffered saline and incubated with goat anti-rabbit horseradish peroxidase-conjugated secondary antibody (1:5,000; Gaithersburg, MD, United States) for 1 h. The expression of the specific protein was visualized using a commercial electrochemiluminescence kit (Invitrogen).

Ultra-performance liquid chromatography was coupled with time-of-flight mass spectrometry analysis (UPCL-TOF/MS^E^).

### Sample Preparation Procedures

Stock standard solutions of glucose (0.1 mol/L) and AOS (10 mg/ml) were prepared. The diluted solutions were separately prepared by mixing 200 μL of stock internal standard solution and AOS, and 200 μL of 0.4 mol/L AEC/NaBH3CN and 40 μL acetic acid. Each homogenized sample was weighed in a 15 ml plastic centrifuge tube and then heated in a water bath (70°C, 60 min). Before the analysis, a few amounts of the extract were diluted in a solution of methanol for preparing the sample solution. Subsequently, a membrane with a pore size of 0.22 μm was used to filter the solution. The volume of the injection was 10 μL.

### Conditions for Performing Mass Spectrometry

The Xevo G2S TOF (Waters MS Technologies, Manchester, United Kingdom) mass spectrometer was used to perform mass spectrometric (MS) analysis and electrospray ionization was performed in the positive ion mode. The capillary voltage reached 3.0 kV. The low and high collision energies were 6 eV and 15–45 eV, respectively. The temperature of the ion source was 100°C. Atomization was performed at 400°C. The flow rate of desolvation reached 600 L/h. Leucine-enkephalin (leu-enk) was used as the lock mass, and the mass axis was corrected with sodium formate.

Spectral data analysis and quantification were completed using Mass Lynx V4.1 and Target Lynx software, respectively. Data acquisition and analyses or quantification were performed using the MassLynx version 4.1 software and the TargetLynx software, which adopt the same standard and calibration model as that of UPLC-QTOF/MS^E^. A calibration curve of *R* ^2^ > 0.99 met the data acceptance criteria.

### Statistical Analyses

The data are presented as the mean ± SD from at least three independent experiments. SPSS 17.0 (SPSS, Inc., Chicago, IL, United States) was used for statistical analysis. Significance was tested by a one-way analysis of variance. Differences between groups were considered statistically significant if the *p-*value was <0.05.

## Results

### Effects of AOS on the RBG Level and Body Mass of Db/Db Mice

**FIGURE 2:**
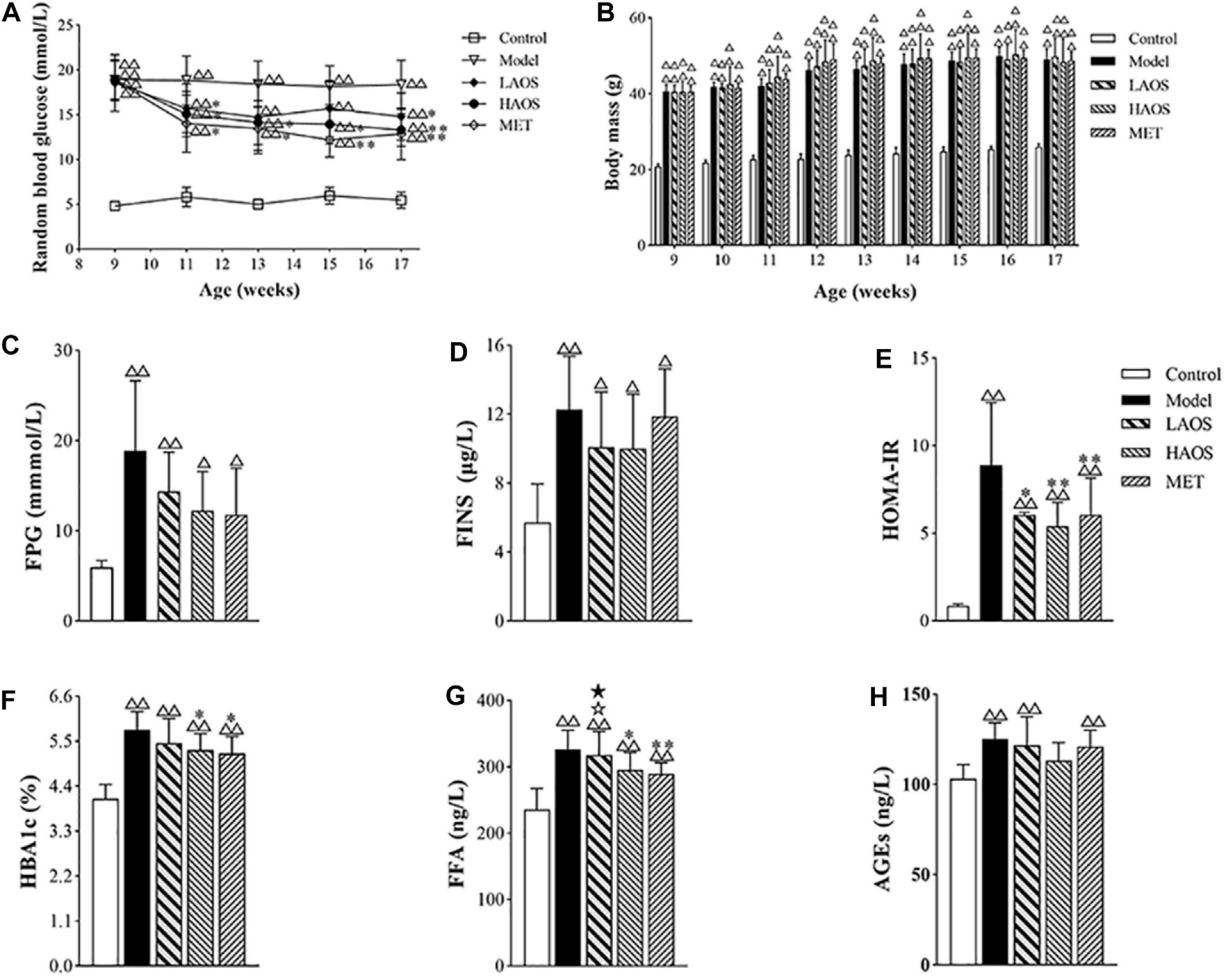
Effects of AOS on glucose metabolism in db/db mice. **(A)** RBG level of the different groups after 8 weeks. **(B)** Body mass of different groups at 9–17 weeks. Distilled water, metformin (MET), or AOS was administered orally to 9-week-old control or model group for 8 weeks, and FPG **(C)**, FINS **(D)**, HOMA-IR index **(E)**, HBA1c **(F)**, FFA **(G),** and AGEs **(H)** were determined. All values are expressed as the mean ± SD (n = 10). ^△△^ *p* < 0.01 versus the control group; *p < 0.05, **p < 0.01 versus the model group. ^☆^ *p* < 0.05 versus MET group; ^★^ *p* < 0.05 versus HAOS group. The values were presented as mean ± SD (n = 10).

The model group showed significantly higher baseline RBG levels than the control group. The RBG levels in the AOS-treated group were significantly lower than those in the model group. After 8 weeks of AOS administration, the RBG levels in the HAOS and LAOS groups markedly decreased (Figure 2A). It was worth noting that the dose of AOS (380 mg/kg/day or 750 mg/kg/day) was determined in our previous study (Bao et al., 2020). As expected, the body weight of the mouse in each group increased gradually (Figure 2B). There was no obvious change in the growth trend during AOS administration, and the growth curve was similar to that of the model group.

### Effects of AOS on Serum Insulin Levels

As shown in Figure 2C–E, the levels of FPG and FINS and the HOMA-IR index were markedly increased in the db/db diabetic mice. Compared with the control group, the FPG and FINS levels and the HOMA-IR index in the AOS-treated groups were significantly lower. The HAOS group particularly showed a notable reduction in the FPG levels (Figure 2C–E). Although, there were no significant differences in FPG and FINS levels between the model and AOS-treated groups, AOS- and MET-treated groups showed a lower HOMA-IR index compared to the model group (Figure 2C,D).

### Effects of AOS on Serum HbA1c, FFA, and AGE Levels in Mice

As expected, the levels of HbA1c, FFA, and AGEs increased in the model group. The HAOS- and MET-treated groups showed lower HbA1c and FFA levels compared to the model group (*p* > 0.05, Figure 2F,G). Especially, the AGE level in the HAOS group was not different compared to that in the control group (Figure 2H). The levels of HbA1c and FFA in the LAOS-treated group were significantly higher than those in the HAOS- and MET-treated groups (Figure 2F,G).

### Effects of AOS on the Pathological Changes in the Pancreas of db/db Diabetic Mice

**FIGURE 3:**
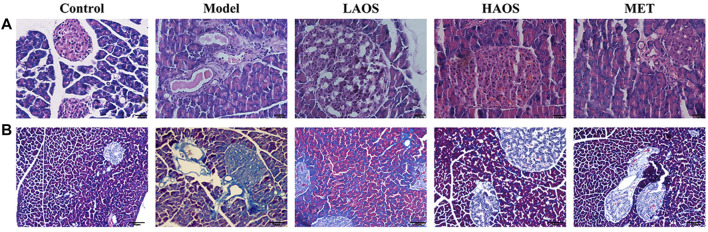
Effects of AOS on the pathomorphism of pancreatic tissues in db/db mice. After sacrifice, the mouse pancreas was removed. Portions of the mouse pancreas from **(A)** were fixed and subjected to H&E staining. Masson trichrome staining **(B)** of the pancreatic tissues was conducted.

We investigated the effects of AOS treatment on the pathological changes in the pancreas of all the groups by performing H&E staining and Masson trichrome staining. H&E staining showed that pancreatic islets in the control group exhibited normal characteristics such as the regular elliptical shape and a clear boundary. Compared to the control group, the number of islets in the model group was markedly lower. The pancreas was severely shrunken and the islets had no clear boundaries. AOS treatment reversed this pathological damage by improving the morphological arrangement of islets and increasing their number, in addition to restoring a clearer structure of the surrounding tissues. In particular, there were more pathological changes in the pancreas of the HAOS group than in the model group (Figure 3A). Next, the results of the Masson trichrome staining indicated that there was no blue collagen fiber in the pancreatic tissues of the control group, the cell contours were clear, the nucleus was centrally located, and the cell boundary was clear. In the model group, the blue-stained collagen fiber was observed between the pancreatic tissues. The blue collagen fiber in the AOS-treated groups was significantly less than that in the model group (Figure 3B).

### Expression of IRS-1, IRS-2, and Glut4 in the Pancreas

**FIGURE 4:**
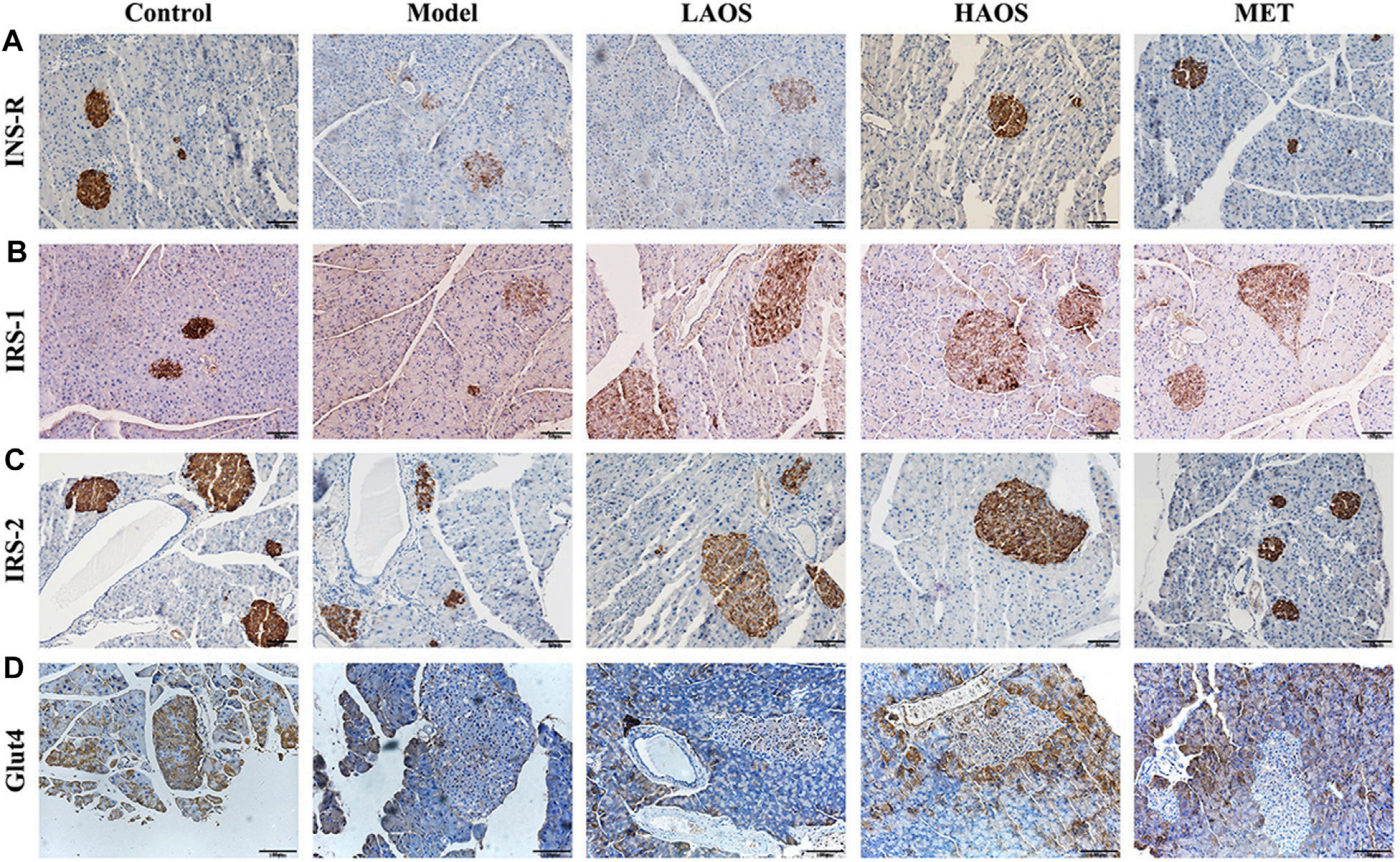
Effect of AOS on INS-R, IRS-1, IRS-2, and Glut4 expression. Wax-embedded sections of db/m and db/db mice pancreas showing the expression of INS-R **(A)**, IRS-1 **(B)**, IRS-2 **(C)**, and Glut4 **(D)** as revealed by immunohistochemistry.

We performed immunohistochemical staining by using antibodies against INS-R, insulin receptor substrates (IRS-1 and IRS-2), and Glut4 to determine the expression of INS-R, IRS-1, IRS-2, and Glut4 in the pancreas (Figure 4). Light staining of INS-R (Figure 4A), IRS-1 (Figure 4B), IRS-2 (Figure 4C), and Glut4 (Figure 4D) was observed in the pancreas of hyperglycemic db/db mice. On the other hand, INS-R, IRS-1, IRS-2, and Glut4 were strongly expressed in the pancreatic tissues of the control group than in the remaining groups.

### Insulin Secretion by MIN6 Cells

**FIGURE 5:**
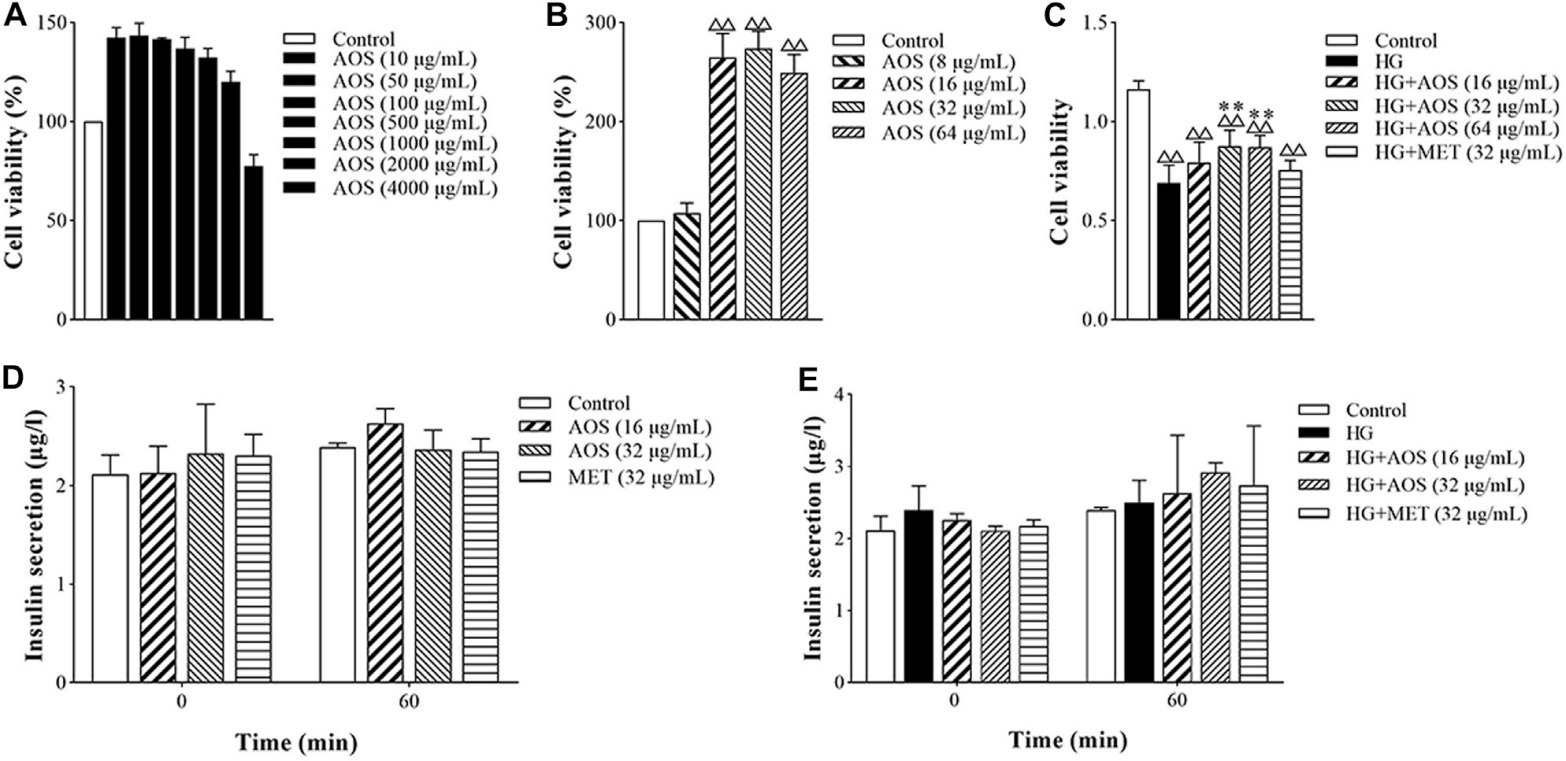
AOS improved the proliferation of pancreatic MIN6 cells. **(A)** MIN6 cells were cultured and treated with different concentrations of AOS (0, 10, 50, 100, 500, 1,000, 2000, and 4,000 μg/ml) for 24 h ^△△^ *p* < 0.01 versus control (0 μg/ml) group. **(B)** MIN6 cells were treated with different concentrations of AOS (0, 8, 16, 32, and 64 μg/ml) for 24 h. **(C)** MIN6 cells were incubated with normal glucose (5.5 mM) or high glucose (33.3 mM) for 24 h, and then the cells were cultured in the absence or presence of AOS with different concentrations (16, 32, and 64 μg/ml) for 24 h ^△△^ *p* < 0.01 versus control (0 μg/ml) group; ^**^ *p* < 0.01 versus the high-glucose group. **(D)** The insulin levels after incubation with AOS for 1 h. **(E)** The insulin levels after incubation with glucose for 1 h.

Cell viability values of MIN6 cells treated with AOS were higher than those of cells without AOS treatment at concentrations of 0–2000 μg/ml (Figure 5A). It was linear in the range of 10–100 μg/ml, and the proliferation activity gradually decreased at 500 μg/ml and was toxic to cells at 4,000 μg/ml. Cell viability was better in the 16, 32, and 64 μg/ml concentration range (Figure 5B). Compared with the control group, high glucose (33.3 mmol/L) concentration significantly decreased the viability of MIN6 cells. AOS addition enhanced the viability of high glucose-treated cells (Figure 5C). In addition, in all the AOS-treated groups, there was no significant effect on insulin secretion by non-hyperglycemic-stimulated MIN6 cells, and there was no difference compared with that in the control group (Figure 5D). The concentrations of 16 and 32 μg/ml AOS promoted the insulin secretion by MIN6 cells stimulated by high glucose concentrations, and the concentration of 32 μg/ml AOS had the most obvious effect. However, there was no statistically significant difference among the groups when the MIN6 cells were incubated under high-glucose or AOS conditions (Figure 5E).

### Apoptosis of MIN6 Cells

**FIGURE 6:**
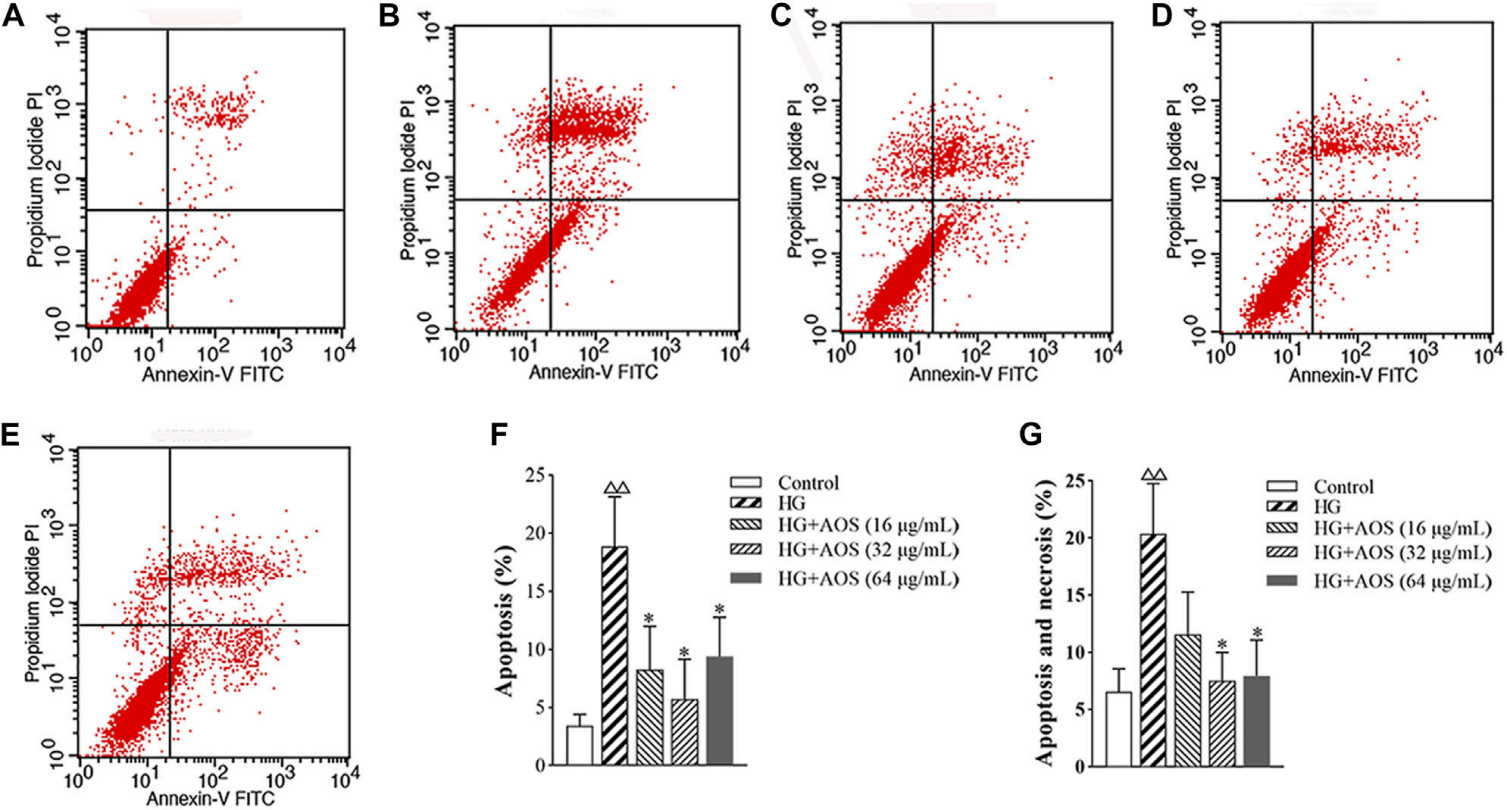
AOS inhibits high glucose-induced MIN6 cell apoptosis. MIN6 cells were preincubated with glucose (33.3 mmol/L) for 24 h and then exposed to AOS (16, 32, and 64 μg/ml) for 24 h. The rate of apoptosis was determined by flow cytometry. **(A)** Control. **(B)** High glucose. **(C)** High glucose + AOS (16 μg/ml). **(D)** High glucose + AOS (32 μg/ml). **(E)** High glucose + AOS (64 μg/ml). (**F)** Quantification of apoptotic cells. **(G)** Quantification of apoptotic and necrosis cells. Values are expressed as the mean ± SD (n = 3). ^△△^ *p* < 0.01 vs. Control; ∗*p* < 0.05 compared with high glucose.

The apoptotic rate of the MIN6 cells at high glucose concentrations was significantly higher than that in the control groups, whereas, after the AOS treatment, the apoptotic rate was lower than that in the high-glucose-treated group. The 32 μg/ml concentration of AOS resulted in a greater reduction in apoptosis than treatment with 64 μg/ml AOS (Figure 6).

### Protein and mRNA Levels in MIN6 Cells

**FIGURE 7:**
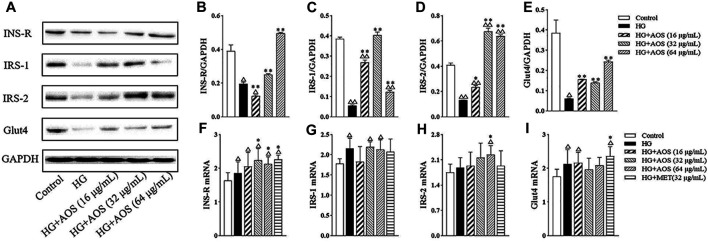
The effects of AOS treatment at the insulin signaling pathway of MIN6 cells. **(A)** Western blotting of INS-R, IRS-1, IRS-2, and Glut4 protein expression in the cells. **(B, C, D, E)** Fold change by the positive control (HG + MET). **(F, G, H, I)** Relative mRNA expression against GAPDH in MIN6 cells. All data are expressed as the mean ± SD. ^△^ *p* < 0.05 or ^△△^ *p* < 0.01 vs. Control; *p < 0.05 or **p < 0.01 vs. high glucose (HG).

As shown in Figure 7A, the protein levels of INS-R, IRS-1, IRS-2, and Glut4 were significantly lower in the high-glucose group than in the control group. However, AOS treatment increased the levels of these proteins. Especially, the AOS-treated (64 μg/ml) group resulted in the highest increase in the protein levels. To further investigate the potential mechanisms of AOS, the mRNA levels of INS-R, IRS-1, IRS-2, and Glut4 in MIN6 cells were determined. Compared with the high glucose-treated group, we found that AOS did not significantly reduce the IRS-1 and Glut4 mRNA levels (Figure 7F,I). High glucose concentration induced a reduction in INS-R and IRS-2 mRNA levels, but AOS reversed these effects by markedly upregulating INS-R and IRS-2 mRNA expression (Figure 7G,H).

### Identification of AOS Constituents by UPLC-Q-TOF/MS^E^ Analysis

**FIGURE 8:**
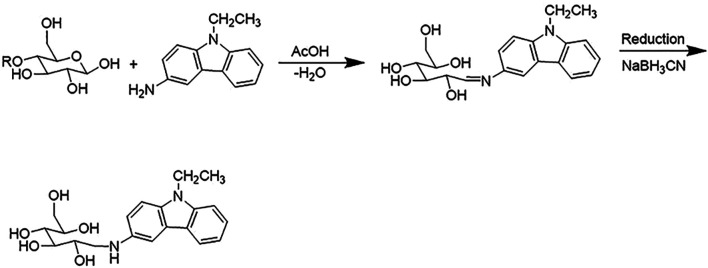
The reaction mechanism of AEC with sugars in AOS.

**FIGURE 9:**
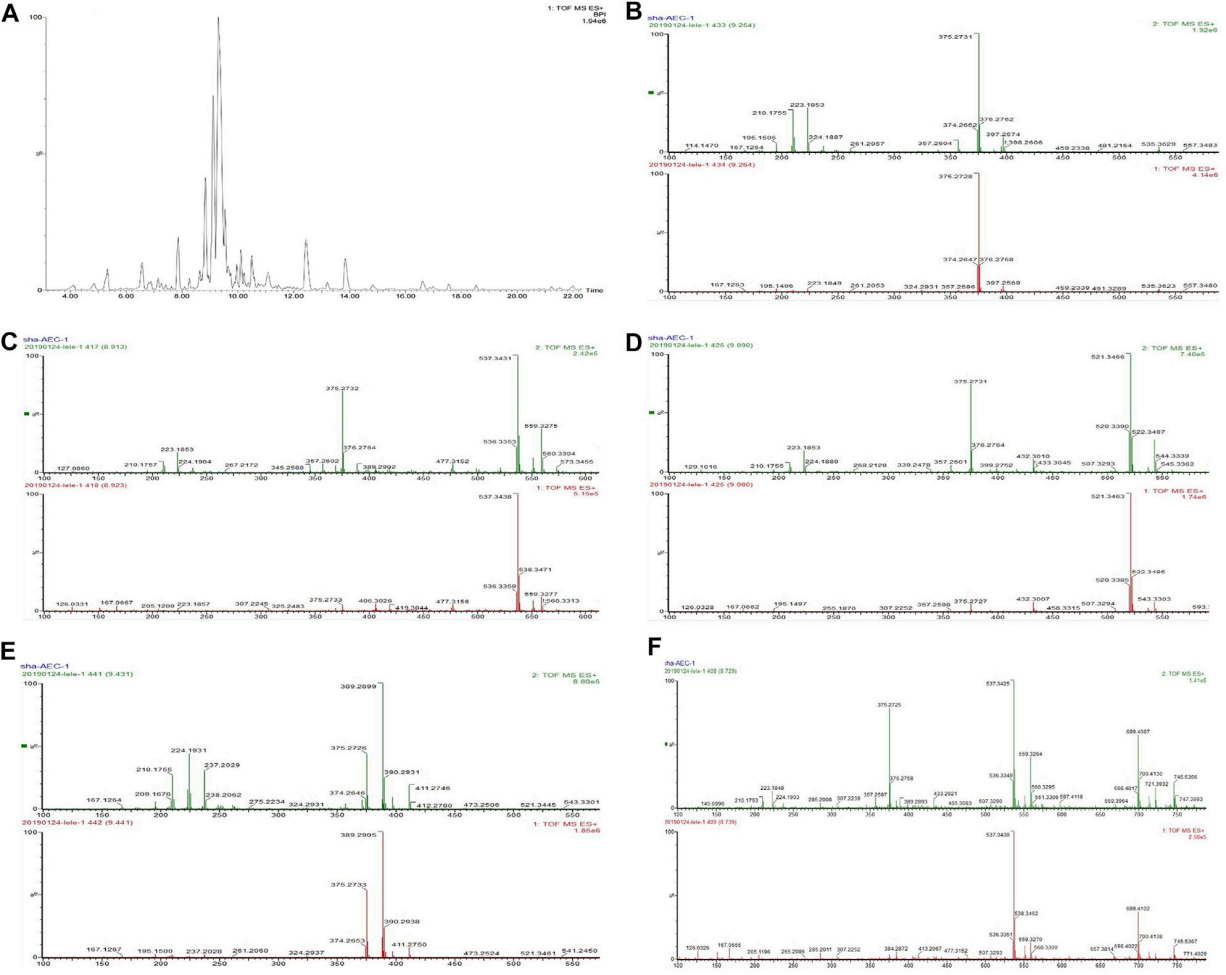
Chromatogram of AOS derivatives of five kinds of monosaccharide (A) and base peak chromatogram of the AOS obtained using an MSE data collection method (UPLC-Q-TOF/MS^E^) in positive mode. **(B)** Glucose, peak 375. **(C)** Lactose, peak 537. **(D)** Rutinose, peak 521. **(E)** Glucuronic acid, peak 389. **(F)** Maltotriose, peak 699.

UPLC-Q-TOF/MS^E^ analysis of AOS was performed with precolumn derivatization with AEC. The reaction mechanism of AEC with sugars in AOS is shown in Figure 8. The enamine was generated by the reaction of the reducing end of AOS and the primary amine of AEC and then reduced to secondary amines by NaBH3CN so that sugars could be labeled by the AEC. The AEC-derived AOS solution was directly separated and analyzed by UPLC-QTOP/MS^E^ to obtain five types of sugars including glucose, lactose, rutinose, glucuronic acid, and maltotriose. These sugars were inferred by the molecular formula by obtaining a chromatographic peak and a fragment ion peak, as well as by referring to databases such as MassBank, Scifinder, and ChemSpider and general literature (Figure 9). Moreover, the contents of these five sugars of AOS were as follows: glucose, 1.38 ± 0.02 mg/g; lactose, 8.08 ± 0.48 mg/g; rutinose, 14.9 ± 0.5 mg/g; maltotriose, 0.12 ± 0.08 mg/g; and glucuronic acid, 15.35 ± 0.49 mg/g (Table 2).

**TABLE 2:** The five sugars contents of AOS.

Sample	Glucose	Lactose	Rutinose	Glucuronic acid	Maltotriose
	(mg/g)	(mg/g)	(mg/g)	(mg/g)	(mg/g)
AOS	1.38 ± 0.02	8.08 ± 0.48	14.9 ± 0.5	15.35 ± 0.49	0.12 ± 0.08

## Discussion

In this study, we determined that orally administered AOS regulated blood glucose levels in db/db mice by improving pancreatic β-cell mass, which in turn contributed to the well-controlled RBG levels observed in AOS-treated db/db mice. Moreover, our study showed that in AOS-treated db/db mice and high glucose-induced MIN6 cells, the expression of INS-R, IRS-1, IRS-2, and Glut4 was upregulated in an insulin-dependent manner.

AOS, extracted from traditional Mongolian medicinal plants, is effective in lowering blood glucose levels, improving glucose tolerance, and alleviating insulin tolerance in T2DM (Bao et al., 2016a; Bao et al., 2016b; Bao et al., 2018). Our previous studies attempted to demonstrate the potential mechanism by which AOS mitigates T2DM. For example, AOS can mediate the IRS2/PI3K/AKT/GSK3β/GLUT4 signaling pathway (Saqier et al., 2019). Furthermore, AOS could play a significant role in improving pancreatic function (Bao et al., 2016b). AOS does not have a negative effect on the liver function of db/db diabetic mice (Bao et al., 2020). T2DM is highly related to insulin resistance. For example, β-cell failure results in hyperglycemia and T2DM (Elghazi et al., 2010). Therefore, in this study, we investigated the effects of AOS on insulin secretion and reduction in blood glucose levels. We mainly focused on the antiapoptotic effects of AOS on pancreatic β-cells and its mechanism *in vitro* and *in vivo*. We demonstrated that the regulatory action of AOS might contribute to the inhibition of β-cell apoptosis and promote islet insulin release in db/db mice and high glucose-exposed MIN6 cells.

Risk factors including obesity, inflammation, IR, and hyperinsulinemia can lead to the development of T2DM. Therefore, the choice of the mouse model is an important consideration when studying the mechanisms related to changes in insulin sensitivity, β-cell proliferation, IR, and glucose homeostasis (Burke et al., 2017). The db/db mice, which have an autosomal recessive mutation in the leptin receptor, are internationally recognized as a T2DM animal model. It has the typical clinical manifestations of diabetes such as hyperphagia, hyperglycemia, and polyuria, and their blood glucose concentrations can be maintained at high levels for a relatively long time (Chen et al., 1996; Sataranatarajan et al., 2016; Yu et al., 2019). Therefore, in this study, we used the db/db mice as a model to determine the effects of AOS on IR *in vivo*. Glycated HbA1c, FPG, or OGTT has been used to diagnose T2DM (Lim et al., 2018). Also, under the condition of long-term exposure to high blood glucose levels, the HbAlc level significantly increases, which leads to vascular endothelial injury and vascular dysfunction, and is the basis of diabetic vascular complications. Therefore, HbA1c is a simple and reliable marker for testing insulin sensitivity in diabetic patients, and the optimum level of HbA1c is 5.6–7% in patients with T2DM (Kim and Jung, 2019). In diabetic db/db mice, AOS improves hyperglycemia. In agreement with a previous study, our study showed that AOS significantly decreased the levels of RBG, blood HbA1c, and FFA compared to those in control db/m mice. In addition, AOS treatment decreased FBG and fasting insulin levels. We found that HAOS clearly decreased blood HbA1c levels. Thus, AOS treatment-mediated decreases in the level of HbA1c may be related to the improvement of IR, decrease in blood glucose, and protection of pancreatic function.

The pathogenesis of T2DM is mainly characterized by IR and a decline in pancreatic function. With the prolonged disease course, the function of islet cells gradually decreases and the secretion of insulin also decreases, which further aggravates T2DM (Akash et al., 2013; Zhuo et al., 2018). HOMA-IR index is the most well-known method that has been proposed for determining IR (Baghbani-Oskouei et al., 2019). The fasting insulin level and HOMA-IR were often used to estimate IR in clinical studies (Wang et al., 2017). The present findings suggested that AOS treatment decreased the HOMA-IR index in the model group. The islets (β cells) are endocrine cells producing insulin for maintaining stable blood glucose levels (Ruan et al., 2018). On the other hand, impaired islet function can reduce the number of islets and their ability to secrete optimum insulin levels, which disturbs optimal blood glucose levels, resulting in the continuous rise of blood glucose levels. Some studies have reported that a decrease of β-cell mass in T2DM contributes to increased cell apoptosis (Butler et al., 2003). In other words, the apoptosis of β cells might be the main mechanism of relative insulin deficiency (Demirtas et al., 2016). Islet cell apoptosis not only destroys islet structure but also leads to a reduction in islet cell apoptosis, which affects islet function. The glucotoxicity of cell apoptosis caused by directly damaging islet cells aggravates IR and impairs insulin secretion stimulated by glucose (Liang et al., 2019). Our cell apoptotic study revealed that AOS reduced β-cell apoptosis in pancreatic MIN6 cells and the pancreatic islets of db/db mice. Also, AOS helped protect β cells from death and degeneration, suggesting that AOS treatment can decrease the apoptosis of pancreatic cells and alleviate IR. Moreover, many animal and human studies have demonstrated that FFAs are key factors responsible for IR (Boden 2003; Makarova et al., 2019; Qureshi et al., 2019). Excessive blood FFA levels can cause IR in peripheral tissues by suppressing muscle glucose uptake and glycogen synthesis and cause β-cell dysfunction. Thus, reducing excessive FFA ameliorates whole-body IR in T2DM (Gong et al., 2019). In our study, AOS administration decreased plasma FFA levels in the AOS-treated mice. In addition, AGEs are oxidant compounds of pathogenic significance that contribute to the worsening of various chronic illnesses such as diabetes and other associated risk factors involved in the development of IR (Chang et al., 2015). Unfortunately, AGEs are involved in mechanisms contributing to IR due to direct modification of insulin that alters insulin action, resulting in impaired glucose uptake, inhibited insulin clearance, or further increased insulin secretion (Nowotny et al., 2015). In the present study, we found that AOS treatment decreased the level of AGEs. Overall, the decrease in FFA and AGE concentrations in response to AOS administration reflects changes in insulin sensitivity.

**FIGURE 10:**
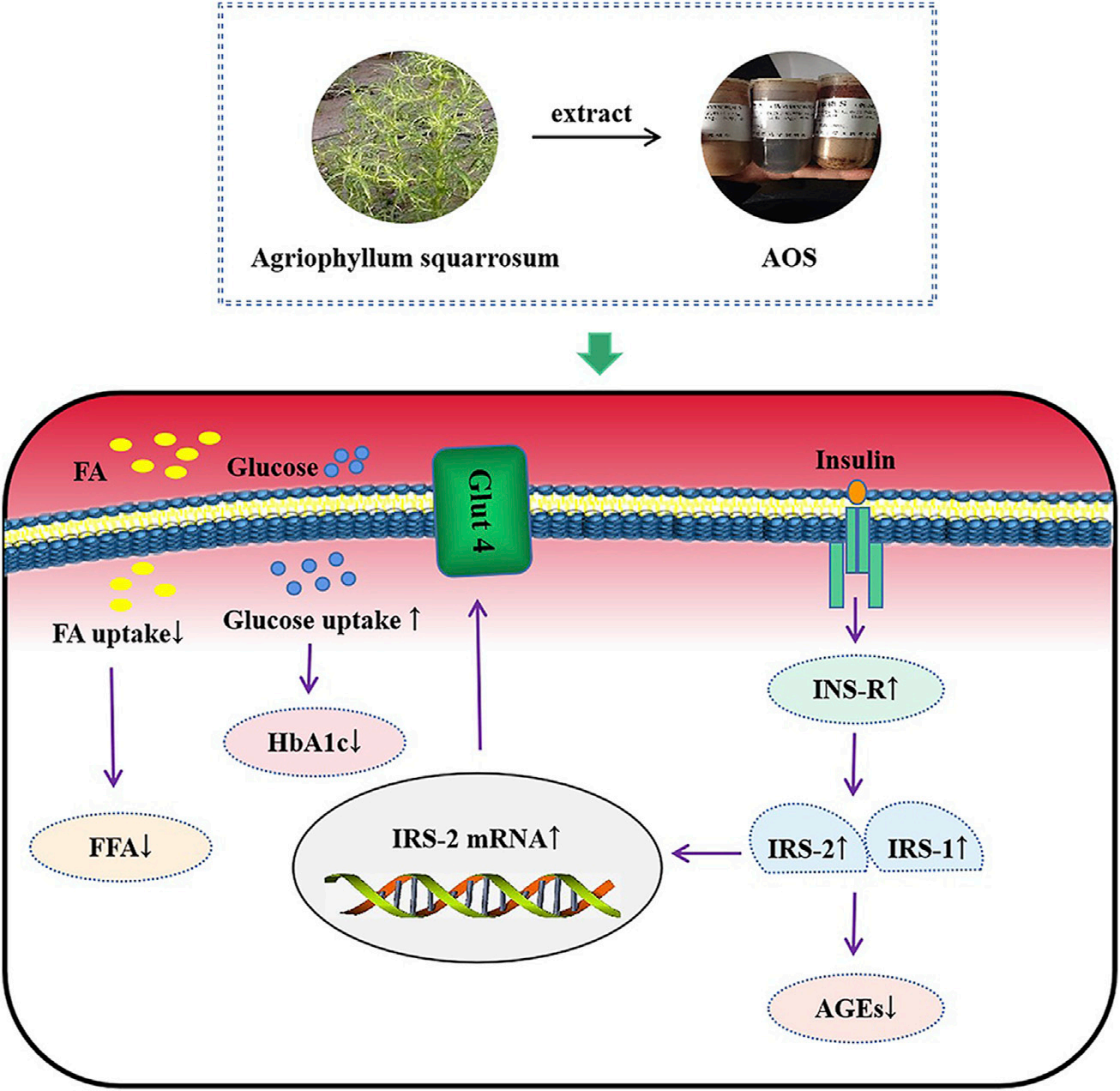
The mechanism diagram of AOS alleviated diabetic insulin resistance in db/db mice and high glucose-induced MIN6 cells.

By performing extensive research on the underlying mechanism of IR, several studies have explained the mechanism of IR, and it is believed that the most important molecular biological cause of IR is blocked or weakened insulin signal transduction (Olefsky and Glass, 2010; Samuel and Shulman, 2016; Li et al., 2018; Peng and He, 2018). Moreover, IRS, which are of great significance for maintaining the growth, division, and metabolism of cells acting as transfer proteins, are important biomolecules in the insulin signaling pathway. IRS-1 and IRS-2 are significant members of the IRS family and are widely expressed in various tissues. They are indispensable cytokines for insulin that play a role in metabolic regulation, maintenance of pancreatic cell function, and promotion of individual growth and development (Ye et al., 2017). These proteins are important insulin pathway-related factors, and the deletion of IRS1 and IRS2 genes leads to the development of diabetes in mice (Wang et al., 2016; Chen Y. et al., 2017; Zhang et al., 2017). Therefore, the maintenance of appropriate levels of IRS-1 and IRS-2 is essential for proper insulin signaling (Jeong et al., 2017). As expected, in diabetic db/db mice and MIN6 cells, high glucose concentrations induced impairment in INS-R, IRS-1, and IRS-2 expression. In the present study, AOS treatment significantly increased INS-R, IRS-1, and IRS-2 expression in db/db pancreatic tissues and MIN6 β cells. These results indicated that AOS may alleviate IR by increasing INS-R/IRS expression (Figure 10).

Glucose uptake is mainly dependent on Glut4, an insulin-regulated transmembrane glucose transporter (Huang et al., 2019; Emamgholipour et al., 2020). Disruption of Glut4 translocation to the cell surface is considered the main cause of IR in T2DM (Abel et al., 2001; Minami et al., 2020; Wang et al., 2020). The control group of db/db mice and MIN6 cells exposed to high glucose concentrations showed decreased levels of Glut4 in pancreatic tissues and MIN6 cells, whereas AOS markedly upregulated Glut4 expression, which possibly accelerated increasing glucose uptake. Thus, AOS improved IR by activating the transmembrane function of Glut4.

The AOS was separated from Mongolia medicinal plants *A. squarrosum*, and our previous research had identified its main active ingredient to be the AOS. Oligosaccharide is composed of a number of condensed monosaccharide molecules, which is a kind of complex and large molecular structure of sugar substances. Meanwhile, the determination of monosaccharide composition in oligosaccharides has been reported to be an important means to explore the structure-activity relationship of oligosaccharides (Di et al., 2003; Langeslay et al., 2012; Sun et al., 2014). After UPLC-Q-TOF/MS^E^ analysis, five sugars including glucose, lactose, rutinose, glucuronic acid, and maltotriose were identified by analyzing the molecular formula obtained in the chromatographic peak. Considering that AOS contains glucose and lactose, the RBG level in mice fluctuated at 15 weeks. Because of the complex composition of AOS and a large number of possible target interactions, AOS may ameliorate T2DM through multiple mechanisms. However, these mechanisms have not been precisely determined yet. Therefore, in our futures studies, we aim to study the individual ingredients and determine their effects on increased blood glucose levels. These future studies will help clarify the mechanisms underlying the beneficial effects of AOS on T2DM and provide an effective drug for treating T2DM.

## Conclusion

To summarize, we reported that AOS treatment significantly improved glucose metabolism in db/db mice and high glucose-exposed MIN6 cells. Furthermore, our results revealed a key mechanism of AOS in regulating IR in the pancreas, wherein the expression of INS-R, IRS-1, IRS-2, and Glut4 was ameliorated. These findings provide a novel mechanistic understanding of the beneficial effects of AOS on IR in the pancreas and can assist in the treatment of IR and T2DM in the future.

Further studies would be required to evaluate the individual ingredients of AOS and determine their effects on regulating the blood glucose levels. These future studies are expected to help clarify the mechanisms underlying the beneficial effects of AOS on T2DM and thereby provide an effective drug for treating T2DM.

## Data Availability

The original contributions presented in the study are included in the article/supplementary material; further inquiries can be directed to the corresponding authors.
